# Does delivery in private hospitals contribute largely to Caesarean Section births? A path analysis using generalised structural equation modelling

**DOI:** 10.1371/journal.pone.0239649

**Published:** 2020-10-08

**Authors:** Rayhan Sk

**Affiliations:** Centre for the Study of Regional Development, School of Social Sciences, Jawaharlal Nehru University, New Delhi, India; Institute of Economic Growth, INDIA

## Abstract

**Background:**

The rate of Caesarean Section (CS) deliveries has shown an alarming rise in recent years. CS is a surgical procedure used when there is apprehension of risk to the life of mother or baby in case of vaginal delivery, but its rates higher than 10–15 per cent are not justifiable. It is well recognised that a CS delivery could have a large number of adverse impacts on women and infants. Several studies, especially in developing countries, have revealed that delivery in private hospitals is one of the most contributing factors in CS deliveries. The present study conceptualises a causal pathway in which the possible risk factors, socio-economic, maternal and pregnancy-related, as well as institutional, influence the chances of CS delivery. It is hypothesised that certain factors would contribute to CS deliveries largely indirectly through the place of delivery, that is, either a public or private institution.

**Methods and findings:**

To test the hypotheses, this study analysed 146,280 most recent live births delivered in hospitals during the five years preceding the fourth round of India’s National Family Health Survey (NFHS-4), carried out during 2015–2016. The analysis, using generalised structural equation modelling (GSEM), revealed that many exogenous variables considered in the path models influence CS deliveries significantly, directly and/or indirectly through the place of delivery factor. Prominent among these are wealth index and receiving ANC services at only private hospitals; the total effects of these variables are even higher than the direct/total effect of place of delivery.

**Conclusion:**

From this finding, it could be said that the place of delivery is a proximate determinant of a CS delivery or a mediator of other co-factors. Interventions to curb higher CS deliveries should be focused on improving the quality of public health sectors and on developing protocols for CS deliveries.

## 1. Introduction

A delivery by Caesarean Section (CS) is a surgical procedure that is used if it is apprehended that a complicated pregnancy or complications arise during labour make vaginal delivery difficult and puts the mother or the foetus at risk. CS delivery may also have adverse impacts on women and infants. A study found that a CS delivery is associated with a higher risk of maternal mortality, ureteral tract and vesical injury, placenta previa, fetal death and uterine rupture in future pregnancies, hysterectomy, abdominal pain and neonatal respiratory morbidity [1]. Another study found that CS delivery is associated with a significantly higher risk of placenta accreta in a future pregnancy [2]. Studies showed that women who are delivering by elective CS have almost three times more chance of maternal death compared to women who are normally delivering [3,4]. A review study by Sandall et al. 2018 [5], published in Lancet, concluded that CS may lead to short-term and long-term effects for children and women’ health [5]. Short-term risks are modified immune development, a higher chance of asthma, atopy and allergy, and reduced intestinal gut microbiome diversity. Long-term risks are late childhood asthma and obesity, and preterm birth in the future pregnancy [5].

In this regard, the World Health Organization (WHO) issued an agreement proclamation in 1985, stating that, "There is no justification for any region to have Caesarean Section (CS) rates higher than 10–15 percent" [6]. There is, however, considerable debate about whether CS rates over, 15 per cent mean an over-utilisation of the procedure. It is true that “as with any surgery, Caesarean sections are associated with short- and long-term risks which can extend many years beyond the current delivery and affect the health of the woman, her child, and future pregnancies. These risks are higher in women with limited access to comprehensive obstetric care” [7].

In spite of WHO’s 1985 recommendation, the rate of CS deliveries has risen alarmingly across the globe. A very recent estimation by Betran et al. (2016) [8] shows that the worldwide CS delivery rate had risen from 6.7 per cent in 1990 to 19.1 per cent in 2014 [8]. The highest absolute rise of 14.6 percentage points (from 6.3 per cent to 20.9 per cent) was exhibited by the less developed countries, followed by the higher developed countries with the absolute increase of 12.7 percentage points (from 14.5 per cent to 27.2 per cent), whereas the rate of CS delivery increased by 4.2 percentage points only (from 1.9 per cent to 6.1 per cent) in the least developed countries [8]. Moreover, Betran et al. reported that in some of the developing countries like the Dominican Republic (56.4 per cent), Brazil (55.6 per cent), Egypt (51.8 per cent), Iran (47.9 per cent) and Turkey (47.4 per cent) a substantial proportion of mothers had given births by CS delivery [8].

In India, according to the national representative survey reports, the national average CS delivery rate has become doubled from nine per cent in 2005–06 to 17 per cent in 2015–16 [9,10]. Further, the latest report shows that there are substantial variations in CS delivery rates across the states as well as major geographical regions of India, 2015–16 [9]. For instance, in 2015–16, Telangana is a state of India, has the highest rate of CS delivery (57.7 per cent), followed by Andhra Pradesh (40.1 per cent), Kerala (35.8 per cent) and Tamilnadu (34.1 per cent). All these four higher prevalence states of CS delivery rates belong to the southern part of India. On the other hand, Nagaland has the lowest CS delivery rate (5.8 per cent), followed by Bihar (6.2 per cent), Meghalaya (7.6 per cent), Rajasthan (8.6 per cent), Madhya Pradesh (8.6 per cent). National survey report of 2005–06 estimated that about 18 per cent and 20 per cent deliveries occurred in public hospitals and private hospitals respectively in India [10]. While the latest survey report of 2015–16 estimated that about 52 per cent and 26 per cent deliveries occurred in public hospitals and private hospitals respectively in India. And, there are also considerable variations in both the places of delivery across the states and geographical regions [9]. For example, in 2015–16, more than 50 per cent deliveries occurred in private hospitals in states like Kerala (61.5 per cent), Telangana (61.1 per cent), Gujarat (56.1 per cent) and in Andhra Pradesh (53.3 per cent), whereas, below 10 per cent deliveries occurred in private hospitals in the states like Jammu and Kashmir, Nagaland, Odisha and Arunachal Pradesh. A recent study by Mohanty et al. (2019) [11] showed that the average out of pocket expenditure (OOPE) was US$365 for a CS delivery in private hospitals, US$94 for a CS delivery in public hospitals, US$160 for a normal delivery in private hospitals and US$30 for a normal delivery in public hospitals in India. The inter-state differences in OOPE for all categories were wide-ranging [11]. And, the differences in the type of delivery, place of delivery and OOPE could be explained by variations in quality, affordability and accessibility of maternal health care services, and by the unequal socio-economic development throughout the country. Ohlan, in his study (2013) [12] found that socio-economic development index was far more and systematically developed for southern India as compared to central and northern India [12]. Further, if the latest DHS’s (Demographic and Health Surveys) wealth index, is a proxy measure of income, considered to show the distribution of income, then a wider inequality among states within and between various regions is observed in India, 2015–16. Broadly, south and west India’s condition is better in comparison to other parts of the country. In contrast, the status of central India and, east and north-east India is poor than their counterparts. In the case of women education, south and west India, as well as north-east India are in a good position as compared to north, central and east India according to the latest DHS, 2015–16 [9].

A review study by Sk and Barua (2018) [13] based on 45 studies in developing as well as in developed countries demonstrated that delivery in private hospitals is one of the most influential risk factors of CS delivery in both the developing and developed countries [13]. However, the influence of private hospitals is relatively more prominent on CS delivery in developing countries than developed ones [13]. This review reported that out of 31 studies in developing countries, in 16 studies the association between type of hospitals and CS deliveries was apparent and delivery in private hospitals was the strongest predictor of CS deliveries in seven studies. Contrary to that, out of 14 studies in developed countries, the association between type of hospitals and CS deliveries was found in only five studies and in none of them did delivery in private hospitals emerge as the strongest factor of CS delivery. Therefore, why private hospitals contribute largely to performing CS deliveries over public hospitals in developing countries is a matter of concern and this phenomenon needs to be investigated. Is it the case that delivery in a private hospital contributes largely to a Caesarean birth? And then, what are the factors influencing the childbirth at a private hospital?

There are several studies, especially in developing countries, which demonstrated that childbirth in a private hospital is one of the most contributing factors in CS deliveries [14–23]. However, these studies did not conceptualise a causal pathway in which the possible risk factors influence CS delivery. Most of these studies estimated the direct effects of predictor variables on the incidence of CS delivery. The indirect effects of several factors such as mother’s education and household income which determine the place of delivery have been ignored, and this could lead to misleading inferences. Studies in India as well as in the South Asian context revealed that wealth status is one of the strongest factors influencing the decision to go to private hospitals over public ones for childbirth [24–28]. Studies also have shown that women with a higher level of education choose private hospitals over public ones for delivering their babies [24–26,29]. Besides this, caste systems or social groups also make a difference in access to private or public hospitals, whereas religious groups do not [28]. Additionally, the place of residence, whether urban or rural, is associated with the use of private hospitals for delivery [26]. The place of antenatal care (ANC) service and health insurance coverage further determine the choice between a private or public hospital for deciding where to give birth [29]. A study from the Indian context revealed that there are huge regional differences in making a choice between a private over a public hospital for child delivery [25]. Therefore, the present study aims to investigate the relationship between socio-economic factors, maternal and pregnancy-related factors, spatial factors, institutional factors, place of delivery and the type of delivery.

A review of the existing literature to find out the significant factors influencing CS delivery revealed that among the socio-economic factors, many studies found that the probability of having CS delivery increases with the increase in the level of maternal education [30–32] as also with the increase in the level of income [33–35]. Urban women tend to have CS delivery more than rural women; [36,37] those belonging to socially advantaged sections of the population are also more likely to have CS deliveries compared to socially disadvantaged sections [23]. Among maternal and pregnancy-related factors, studies have shown that the probability of having a CS delivery increases with the increase in maternal age [22,31,38] and also with the increase in a mother’s body mass index; [11,39,40] but the likelihood of having a CS delivery decreases with the increase in birth order [17,41,42]. Extreme low and high birth weight babies are more likely to be delivered by CS [18,43]. The likelihood of a CS delivery increases with the increase in the number of ANC visits; [30,44] breech presentation during delivery is also positively associated with CS delivery [23,45]. Among institutional factors, studies revealed that the type of hospitals and place of ANC visits play a vital role in CS delivery. Those delivering in private health facilities have higher chances of undergoing CS than those delivering in public hospitals [15,21,23,46]. Receiving ANC services at private hospitals is also positively associated with CS delivery [47,48]. Women who have health insurance are more likely to have a CS delivery than women who do not have any health insurance [49,50].

### 1.1. Hypothesised causal pathway

**Fig 1:**
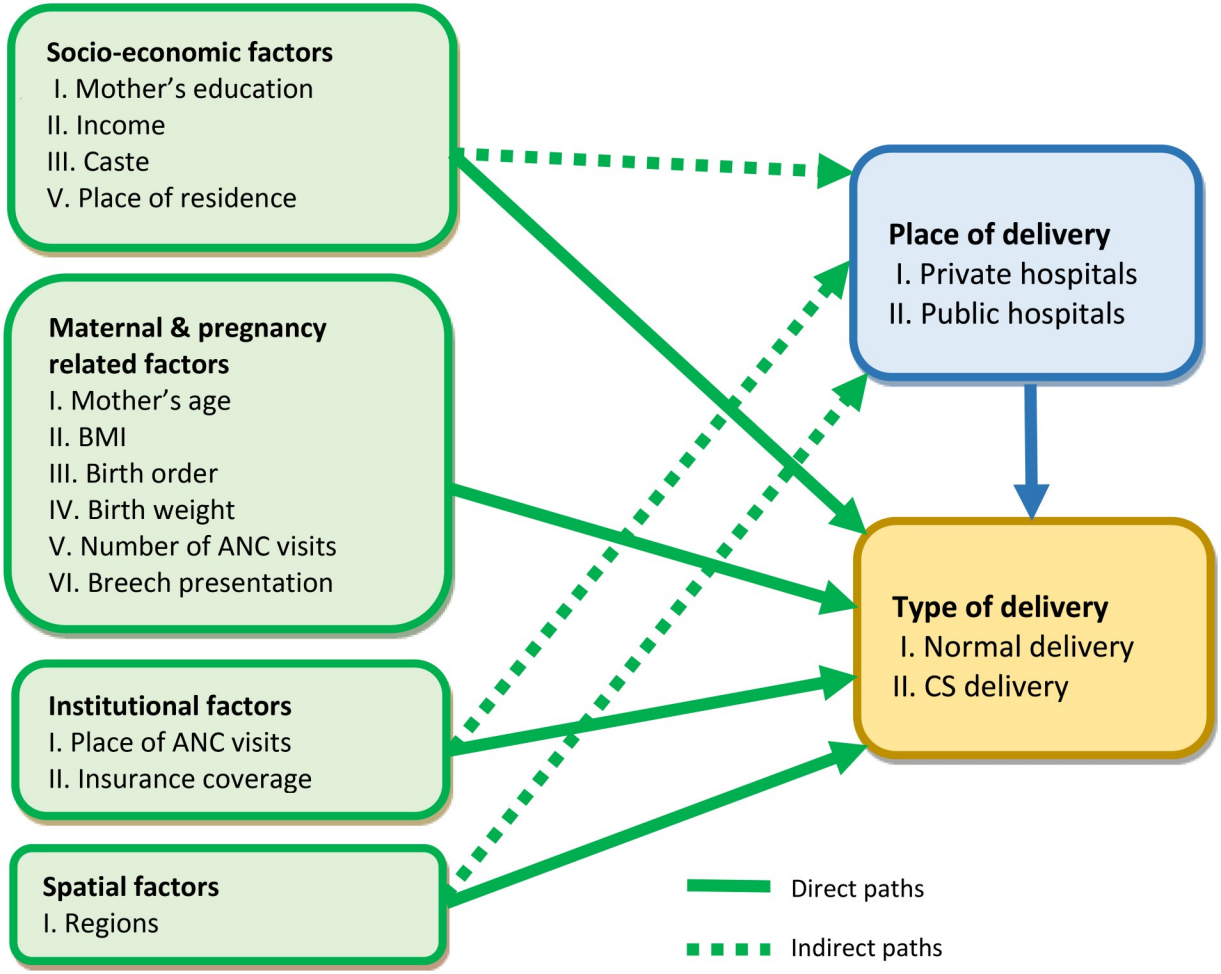
Diagrammatic presentation of the hypothesised causal pathway.

The present study postulates a relationship between socio-economic characteristics, maternal and pregnancy-related factors, institutional factors and spatial factors as exogenous factors, the place of delivery (public hospitals/private hospitals) as an endogenous variable, and the type of delivery as an outcome measure, as shown in Fig 1. It posits that the socio-economic characteristics (education, income, caste or social groups and place of residence), institutional factors (place of ANC visits and insurance coverage), and spatial factors (regions) plausibly affect the place of delivery directly, and further affect the type of delivery directly or indirectly through the place of delivery. Moreover, maternal and pregnancy-related factors, e.g., maternal age, mother’s body mass index (BMI), birth order, birth weight, number of ANC visits and breech presentation possibly affect the type of delivery directly. This study hypothesises that certain socio-economic characteristics (e.g., mother’s education, income) and institutional factors (e.g., place of ANC visits) would contribute to CS delivery largely indirectly through the place of delivery since the accessibility to the private sector hospitals for delivery itself possibly depends on socio-economic factors.

## 2. Methods

### 2.1. Data

The data for this study has been derived from the fourth round of the National Family Health Survey (NFHS-4), which is part of the worldwide Demographic and Health Surveys (DHS), carried out from 2015 to 2016 in India. The National Family Health Survey was a countrywide survey and covered all the 640 districts of India [9]. This survey was designed to gather information at the district level on different aspects of women’s health care utilisation for Reproductive & Child Health (RCH) including access to health facilities. NFHS-4 surveyed a sum of 601,509 households and 699,686 ever-married women of age 15–49 years in India. Data for the most recent live births given by ever-married women of age 15–49 years during the five years preceding the survey were extracted from the files; in the sample, there were 184,641 women who had given a birth during the five years preceding the survey. Since this study’s outcome interest is CS delivery, which is only possible in hospitals (public or private), the present study analysed data on 146,280 (n = 147,002, weighted) of these births delivered in hospitals. The NFHS-4 is the latest round, the data of which is in the public domain. All the data that have been used for this study are fully anonymised.

### 2.2. Outcome variable

The outcome variable was Caesarean section delivery, a dichotomous variable coded as "1" for YES and "0" for NO. Simply put, those women aged 15–49 years who delivered their last live birth by surgical procedure are coded as "1" and those women aged 15–49 years who delivered their last live birth by natural process/vaginally or with assistance or instrument are coded as "0".

### 2.3. Endogenous variable

The place of delivery (public health facility or private health facility) was considered as an endogenous variable since it could be influenced by exogenous factors and at the same time, it could be a risk factor of the CS delivery. The place of delivery is coded as "1" for private hospitals and as "0" for public hospitals.

### 2.4. Exogenous variables

The socio-economic factors included in the analysis are: mother’s years of education (continuous), household’s wealth index scores (continuous), caste/tribe (categorised: others, other backward class/OBC, Scheduled Caste/SC, Scheduled Tribe/ST), and place of residence (dichotomous: rural and urban). Wealth index is a proxy measure of income. Data on income was not available in the NFHS, but the survey had collected information on ownership of assets and housing conditions and constructed an index, a composite measure called wealth index scores, given in NFHS-4 [9,51]. The caste-tribe variable is used since in India social status is often associated with caste membership and certain social groups are recognised according to caste/tribe membership. These are: Scheduled Castes (SC) which traditionally had the lowest social standing; Scheduled Tribes (ST), a group of tribes who were isolated; and other backward castes (OBC), who are identified as backward but did not suffer from discrimination like the SCs and STs; and ‘others’, the rest of the population. Among maternal and pregnancy-related factors; maternal age at last birth (continuous), number of ANC visits (continuous), birth order (continuous), mother’s BMI (categorised: normal, underweight, overweight, obese), weight at birth (categorised: 2500–3999 g, <2500 g, >4000 g, not weighted/don’t know), breech presentation during delivery (dichotomous: no, yes) were taken. Among institutional factors, the place of ANC services (categorised: no ANC/ANC at home, only public hospitals, only private hospitals, public & private hospitals), and coverage by health insurance scheme (dichotomous: no, yes) were considered. Regions are taken from the spatial factors as India is a vast country and has diversity in its geography, culture, socio-economy and health infrastructure. Accordingly, states/Union Territories have been grouped into six major geographical regions: northern India, southern India, western India, central India, eastern India, and north-eastern India). Northern India covers Jammu & Kashmir, Himachal Pradesh, Uttarakhand, Haryana, Punjab, Chandigarh, Delhi, Rajasthan and Uttar Pradesh. Southern India comprises Karnataka, Kerala, Tamil Nadu, Andhra Pradesh, Telangana, Puducherry, Andaman & Nicobar Island and Lakshadweep. Western India includes Gujarat, Maharashtra, Goa, Daman & Diu and Dadra & Nagar Haveli. Central India comprises Chhattisgarh and Madhya Pradesh. Eastern India includes Bihar, Jharkhand, Odisha and West Bengal. North-eastern India covers Arunachal Pradesh, Assam, Manipur, Meghalaya, Mizoram, Nagaland, Tripura and Sikkim. The first category of each categorical variable was considered as the reference category in the path analysis.

### 2.5. Statistical analysis

Some descriptive statistics were computed to understand the sample characteristics, and the differences in per cent of CS delivery by women’s background characteristics are gross differentials obtained through bivariate analysis. This study employed generalised structural equation modelling (GSEM) to test the hypothesised causal pathway by which a mother’s education, income, and place of ANC services would contribute to CS delivery largely indirectly through the place of delivery. This method lets us set up and test a plausible path model through which factors are linked to CS delivery. The “gsem” command used in STATA (v. 13.1), which fits generalised structural equation models to estimate the direct and indirect effects of socio-economic factors, maternal and pregnancy-related factors, institutional factors and spatial factors on CS delivery simultaneously [52]. All variables included in the model were observed variables (none are calculated as latent). Structural equation modelling allows one to produce a visual illustration of the model and to estimate a series of models to obtain direct, indirect, and total effects of exogenous variables on the outcome of interest. Generalised structural equation modelling relaxes the constraints of structural equation models (SEM) by allowing for continuous, binary, ordinal, count, and multinomial modelling of dependent variables and allows for interactions between independent variables. SEM cannot demonstrate causality, and the results should be interpreted as correlations; however, it does allow one to determine whether the hypothesised causal pathway is plausible or not [53]. Nonetheless, there are some disadvantages in GSEM. For examples, it does not provide standardised coefficients, goodness of-fit statistics, models fit using summary statistic data [54]. The following formula has been used to compute the indirect effect and total effect of variable “k” on the outcome variable using “nlcom” command:

$$Indirect\;effect=\beta_{12\;}\beta_{k1}$$

$$Total\;effect=\beta_{k2}+\beta_{12\;}\beta_{k1}$$

Where,

*β*_12_ = *path coeffcient from endogenous variable to the outcome* variable

*β*_k1_ = *path coeffcient from exogenous variable* k *to the endogenous* variable

*β*_k2_ = *path coeffcient from exogenous variable* k *to the outcome* variable

*If an exogenous variable is not postulated to inference the endogenous variable*, *then obviously there is no indirect effect*, *and the total effect is simply the direct effect β*_*k*2_.

## 3. Results

### 3.1. Place and type of delivery in India

**Fig 2:**
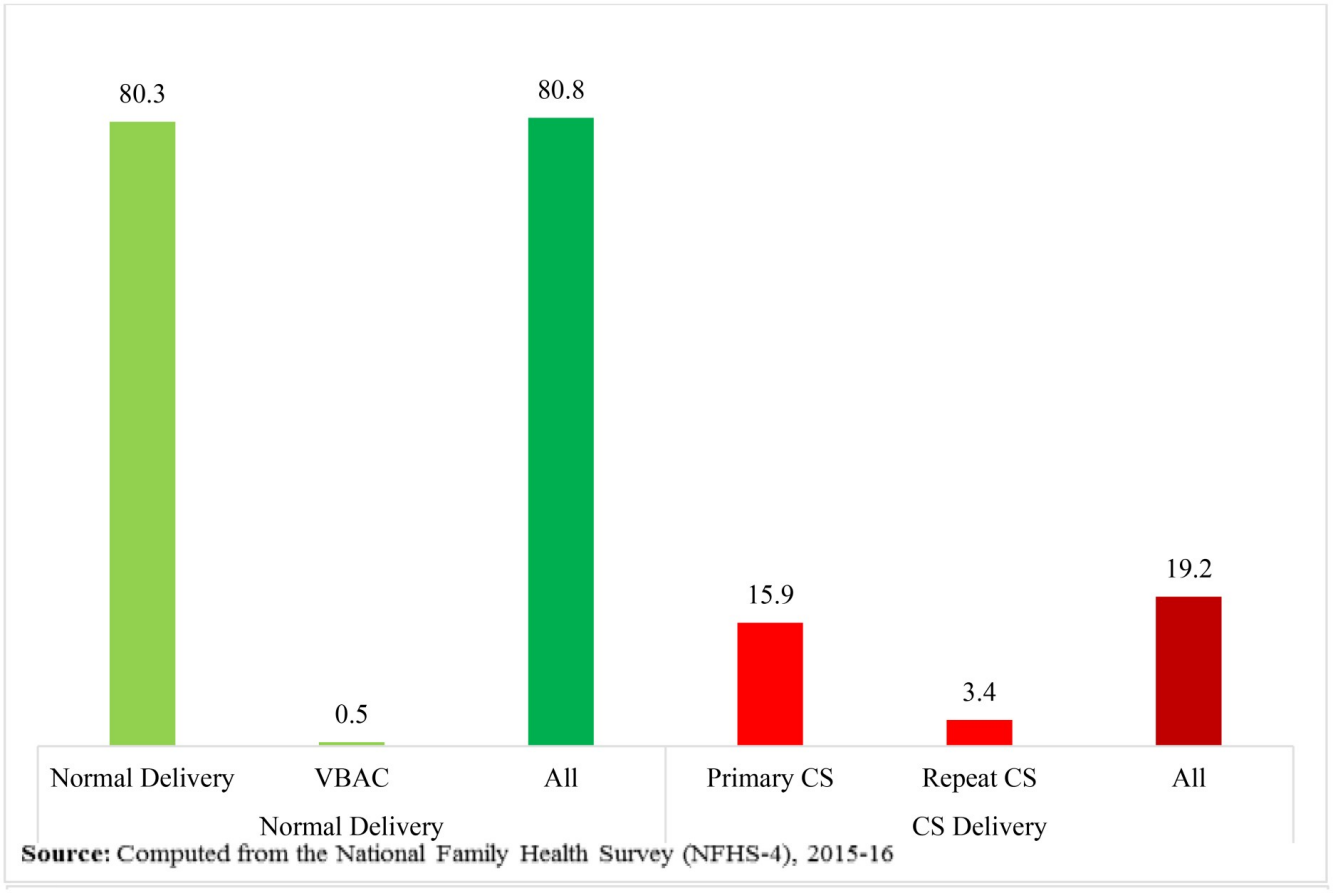
Percentage distribution of type of delivery of last birth during five years preceding the survey, NFHS-4.

**Fig 3:**
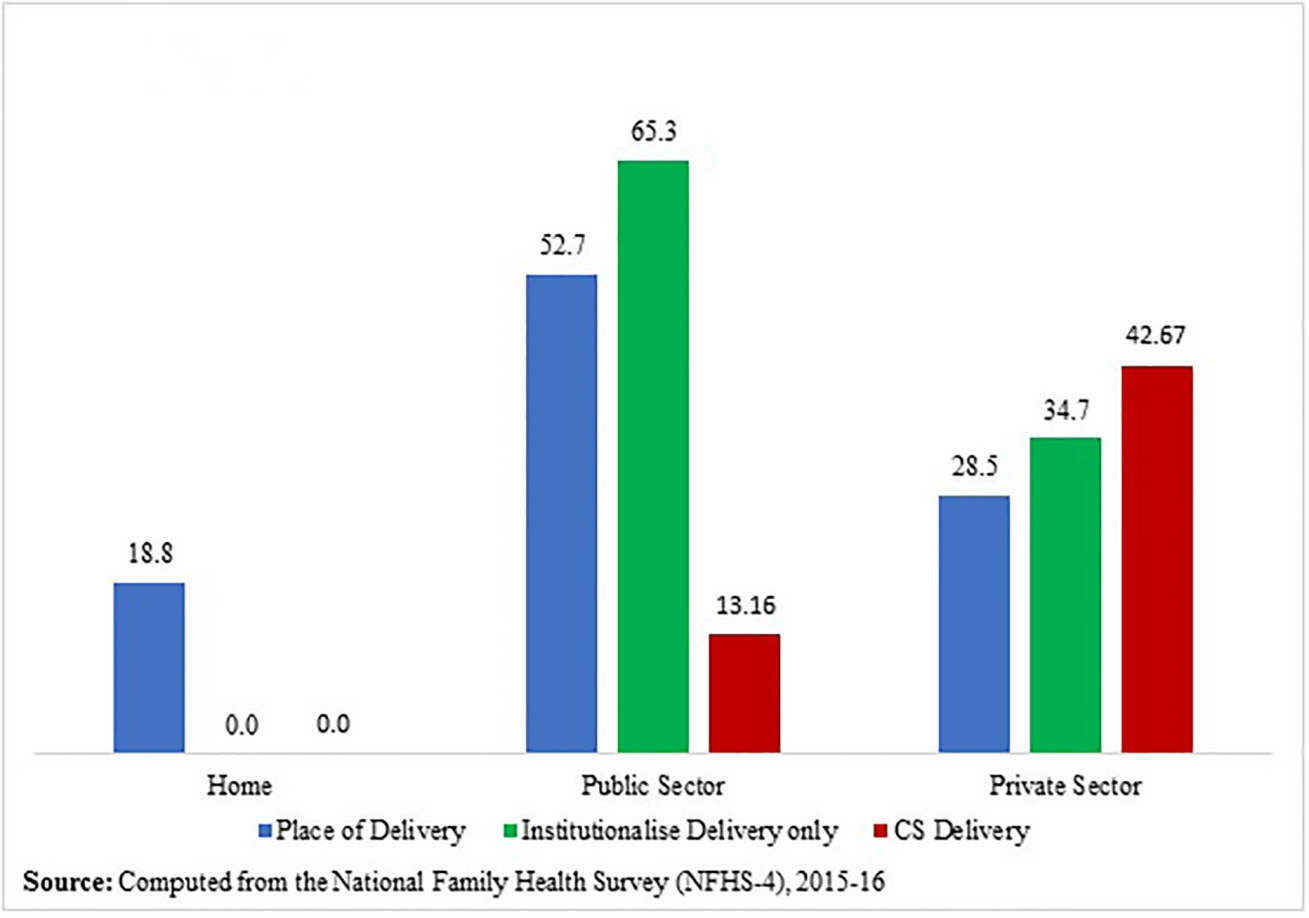
Percentage distribution of place of delivery and CS delivery of last birth during five years preceding the survey, NFHS-4.

In the NFHS-4 sample, 184,641 ever-married women of age 15–49 years had given at least one birth during the five years preceding the survey; the most recent birth has been included in the analysis. Among these births, 19.2 per cent (35,497) were born by CS delivery, in which 15.9 per cent (29,287) and 3.4 per cent (6,210) were primary CS and repeated CS respectively, as shown in Fig 2. Also, Fig 2 exhibits that 80.8 per cent (149,144) births were born normally/vaginally, in which 80.3 per cent (148,224) and 0.5 per cent (920) were normal delivery and VBAC (vaginal birth after caesarean) respectively. So, the repeat CS rate was 87.1 (6,210/7,130*100), while the VBAC rate was 12.9 (920/7,130*100). Fig 3 shows that most of the children (about 81%) were born in institutions during the five years before the survey; among them, 65.3 per cent were delivered in public hospitals and 34.7 per cent were delivered in private hospitals. About 13 per cent children, that is, almost equal to one child in eight children, were delivered by CS in public hospitals, whereas in the case of private hospitals, about 43 per cent children, in other words, almost two children in five children were delivered by CS. The overall institutional CS delivery rate for the last birth was 23.4 per cent in India. Of these births, 63.3 per cent and 36.7 per cent occurred in private and public hospitals respectively. On the other hand, about 19 per cent of children were delivered at home or elsewhere.

### 3.2. Descriptive statistics

The final study sample included 146,280 most recent live births which took place in hospitals during the five years preceding the survey. Table 1 presents the descriptive statistics of the study population and the results of the bivariate analysis. The average age of mothers was 25.4 years [SD (standard deviation) = 4.84], the mean birth order was 2.16 (SD = 1.32), the mean number of ANC visits was 4.83 (SD = 4.61) and the average years of a mother’s education was 7.3 years (SD = 5.11). Higher variations in CS delivery rates are found among the categories of mother’s body mass index (BMI), place of residence, place of ANC visits and caste groups. In addition, a wider variation in CS delivery rate is also observed across the six major geographical regions. The highest CS delivery rate is found in southern India, followed by western India and north-eastern India.

**Table 1:** Descriptive statistics of women who had given last live birth at hospitals during the last five years preceding the survey, NFHS-4, India.

Continuous variables						
**Variables**	**Sample**	**Mean**	**Std. Dev**.	**Min**	**Max**	
Mother’s age	146280	25.40	4.84	12.92	48.58	
Birth order	146280	2.16	1.32	1.00	15.00	
Number of ANC visits	146280	4.83	4.61	0.00	30.00	
Mother’s years of education	146280	7.32	5.11	0.00	20.00	
Wealth index	146280	6558.08	96806.15	-231915.00	290981.00	
Categorical variables						
	Unweighted			Weighted		
**Variables**	**Sample**	**Per cent**	**CS rate**	**Sample**	**Per cent**	**CS rate**
**Weight at birth**						
2500–3999 g	107,137	73.2	20.3	107,904	73.4	24.2
<2500 g	22,530	15.4	21.6	23,510	16	25.1
>4000 g	4,876	3.3	27.1	4,591	3.1	29.5
Not weighted	11,737	8	10.7	10,997	7.5	9.7
**BMI**						
Normal	89,728	61.4	18	87,680	59.7	21.2
Underweight	31,813	21.8	12.1	32,957	22.4	14.7
Overweight	19,576	13.4	34.7	20,585	14	39.2
Obese	5,115	3.5	46.9	5,706	3.9	50.4
Missing	48			74		
**Health insurance**						
No	124,332	85	19.5	123,010	83.7	22
Yes	21,948	15	22.6	23,993	16.3	30.7
**Place of residence**						
Rural	104,941	71.7	16.3	99,098	67.4	18.7
Urban	41,339	28.3	29.1	47,904	32.6	33.2
**Caste group**						
Others	35,563	24.3	27.8	37,955	25.8	29.8
Other Backward Class (OBC)	59,383	40.6	19.6	65,078	44.3	23.2
Scheduled Caste (SC)	27,292	18.7	16.9	30,842	21	20.1
Scheduled Tribe (ST)	24,042	16.4	12.7	13,127	9	13.6
**Breech presentation**						
No	128,417	87.8	19.4	126,743	86.2	23.1
Yes	17,863	12.2	24.3	20,259	13.8	25.3
**Place of ANC visits**						
No ANC/at home	25,647	17.5	13.0	26,739	18.2	15
Only public hospitals	77,741	53.2	15.2	69,162	47.1	17.2
Only private hospitals	31,647	21.6	34.4	38,615	26.3	37.8
Public & private hospitals	11,245	7.7	27.9	12,486	8.5	31.3
**Regions**						
North India	49,697	34	18.5	41,661	28.3	17.4
South India	18,779	12.8	36.3	32,191	22	39.5
West India	12,144	8.3	21.3	21,541	14.7	23.3
Central India	18,898	12.9	12.7	13,087	9	13
East India	28,159	19.3	16	33,486	22.8	20
North-east India	18,603	12.7	19.9	5,037	3.4	21.4
**All**	**146,280**	**100**	**20**	**147,002**	**100**	**23.4**

**Source**: Computed from the National Family Health Survey (NFHS-4), 2015–16.

### 3.3. Estimated path’s coefficients using generalised structural equation modelling

**Fig 4:**
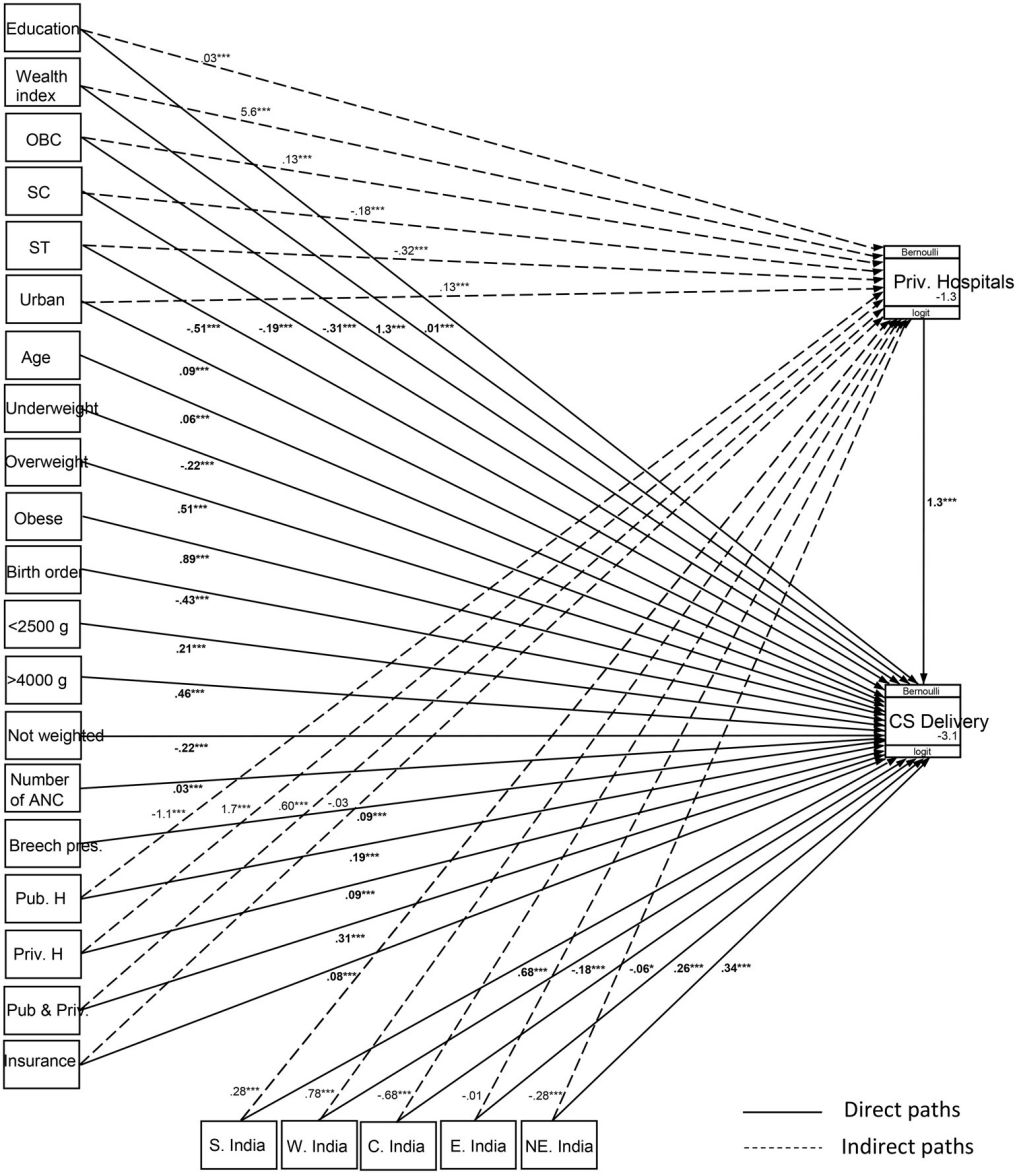
Path analysis using generalised structural equation modelling.

Fig 4 presents the results of path analysis, in which solid arrow lines indicate the direct paths and dashed arrow lines indicate the indirect paths, and the values with bold fonts are the coefficients of directs paths; the coefficients have also been shown in Table 2 along with the *p*-values and confidence intervals. From Fig 4 and Table 2, it may be seen that all the exogenous variables and the endogenous ones considered in the path model influence CS delivery significantly directly or indirectly or in both the ways. However, the magnitude of effects varies considerably across the paths. The figure shows that the direct path coefficient values for the place of birth (B = 1.31, *p*<0.0001) and for wealth index (B = 1.27, *p*<0.0001) are quite a bit higher than the other direct paths linked to CS delivery. Also, it should be noted that the direct path coefficient of place of birth is the highest in the model. On the other hand, in the case of indirect paths linked to endogenous variable or place of delivery, the wealth index has the highest coefficient (B = 5.57, *p*<0.0001), being followed by receiving ANC service at only private hospitals (B = 1.72, *p*<0.0001). As compared to a mother’s normal body mass index, overweight (B = 0.51, *p*<0.0001) and obese (B = 0.89, *p*<0.0001) mothers also have higher direct path coefficients connected to CS delivery in the model and within the categories as well. The direct path coefficients for outer ranges of normal birth weight (2500–3999 g) also show relatively higher effects on CS delivery. With respect to the privileged community [i.e. general category (others)], the deprived communities [i.e. other backward classes (OBC)] to the most deprived communities [i.e. Scheduled Tribes (ST)] have negative direct effects on CS delivery. Moreover, in the case of indirect paths linked to place of delivery, Scheduled Castes (SC) and Scheduled Tribes (ST) are negatively related to accessibility to private hospitals, but other backward classes (OBC) are positively related to the same. From the direct path coefficients of birth order and mother’s age at last birth, an inverse relationship is observed between the birth order and CS delivery, while maternal age at last birth is positively correlated with a CS delivery. Further, a lower level of direct positive association is found between breech presentation during delivery and CS delivery. Among geographical regions, as compared to northern India, southern India, eastern India and north-eastern India have positive direct effects on CS deliveries, while western India and central India have negative direct effects on the same. Furthermore, indirect paths of western and southern India connected to the place of delivery have positive influences on the place of delivery (taking place in private hospitals). But central India, eastern and north-eastern India have negative coefficients for indirect paths.

**Table 2:** Estimated path’s coefficients of exogenous and endogenous variables using generalised structural equation modelling.

Variables	Coefficients	*p*-value	[95% Conf. Interval]
***Dependent variable*: *CS Delivery***			
**Continuous variables**			
Mother’s age	0.06	<0.0001	0.06–0.06
Birth order	-0.43	<0.0001	-0.45–0.41
Number of ANC visits	0.03	<0.0001	0.02–0.03
Mother’s years of education	0.01	<0.0001	0.00–0.01
Wealth index	1.27	<0.0001	1.04–1.50
**Weight at birth**			
2500–3999 g^®^			
<2500 g	0.21	<0.0001	0.17–0.25
>4000 g	0.46	<0.0001	0.39–0.54
Not weighted	-0.22	<0.0001	-0.29–0.15
**Health insurance**			
No^®^			
Yes	0.08	<0.0001	0.04–0.12
**Place of residence**			
Rural^®^			
Urban	0.09	<0.0001	0.06–0.13
**Caste group**			
Others^®^			
Other Backward Class (OBC)	-0.31	<0.0001	-0.35–0.28
Scheduled Caste (SC)	-0.19	<0.0001	-0.23–0.14
Scheduled Tribe (ST)	-0.51	<0.0001	-0.56–0.46
**Breech presentation**			
No^®^			
Yes	0.09	<0.0001	0.04–0.13
**BMI**			
Normal^®^			
Underweight	-0.22	<0.0001	-0.26–0.18
Overweight	0.51	<0.0001	0.47–0.55
Obese	0.89	<0.0001	0.83–0.96
**Place of ANC visits**			
No ANC/at home^®^			
Only public hospitals	0.19	<0.0001	0.14–0.24
Only private hospitals	0.09	<0.0010	0.04–0.14
Public & private hospitals	0.31	<0.0001	0.25–0.38
**Place of delivery**			
Public hospitals^®^			
Private hospitals	1.31	<0.0001	1.27–1.34
**Regions**			
North India^®^			
South India	0.68	<0.0001	0.63–0.72
West India	-0.18	<0.0001	-0.23–0.12
Central India	-0.06	<0.0340	-0.11–0.00
East India	0.26	<0.0001	0.21–0.30
North-east India	0.34	<0.0001	0.29–0.40
***Endogenous variable*: *Place of delivery***			
**Continuous variables**			
Mother’s years of education	0.03	<0.0001	0.02–0.03
Wealth index	5.57	<0.0001	5.35–5.79
**Health insurance**			
No^®^			
Yes	-0.03	<0.2470	-0.07–0.02
**Place of residence**			
Rural^®^			
Urban	0.13	<0.0001	0.10–0.16
**Caste group**			
Others^®^			
Other Backward Caste (OBC)	0.13	<0.0001	0.09–0.16
Scheduled Caste (SC)	-0.18	<0.0001	-0.23–0.14
Scheduled Tribe (ST)	-0.32	<0.0001	-0.37–0.26
**Place of ANC visits**			
No ANC/at home^®^			
Only public hospitals	-1.09	<0.0001	-1.13–1.05
Only private hospitals	1.72	<0.0001	1.68–1.76
Public & private hospitals	0.60	<0.0001	0.55–0.65
**Regions**			
North India^®^			
South India	0.28	<0.0001	0.23–0.32
West India	0.78	<0.0001	0.73–0.83
Central India	-0.68	<0.0001	-0.73–0.62
East India	-0.01	<0.5460	-0.06–0.03
North-east India	-0.28	<0.0001	-0.33–0.22

^**®**^ = Reference category.

**Source**: Computed from the National Family Health Survey (NFHS-4), 2015–16.

### 3.4. Direct effects, indirect effects and total effects on CS delivery

On the basis of the path coefficients shown in Table 2, the direct effects, indirect effects and total effects of the explanatory variables have been computed and presented in Table 3. It may be noted that the total effects (direct+indirect) of wealth index (B = 8.56, *p*<0.0001) and receiving ANC services only at private hospitals (B = 2.34, *p*<0.0001) are fairly higher on the incidence of CS delivery than the direct effect of place of delivery (B = 1.31, *p*<0.0001) and other covariates. Expectedly, the estimated total effect of wealth index on CS delivery is very high, almost six and a half times that of place of delivery. However, wealth index’s indirect effect (B = 7.29, *p*<0.0001) is nearly five and half times larger than its direct effect (B = 1.27, *p*<0.0001) on CS delivery mediated through place of delivery. And the same phenomena is also witnessed in the matter of place of receiving ANC services at only private hospitals. Likewise, the total effect of receiving ANC services at only private hospitals on CS delivery is almost double that of place of delivery. But the indirect effect of receiving ANC services at only private hospitals (B = 2.25, *p*<0.0001) is much higher than its direct effect (B = 0.09, *p*<0.0001) on CS delivery. Furthermore, in comparison to not receiving ANC services or receiving them at home, taking ANC services from both the public and private hospitals have a positive total effect (B = 1.09, *p*<0.0001) on CS delivery, and that is also mostly mediated by place of delivery. While receiving ANC services at only public hospitals has a negative indirect effect and total effect on CS delivery, it has a small positive direct effect on CS delivery. The educational level of mothers also shows a significant positive total effect on CS delivery at a lower level in the hypothesised causal pathway.

**Table 3:** Estimated direct effects, indirect effects and total effects of exogenous and endogenous variable on CS delivery.

Variables	Direct effects B [95% CI]	Indirect effects B [95% CI]	Total effects B [95% CI]
***Exogenous variables***			
**Continuous variables**			
Mother’s age	0.06***[0.06–0.06]	- -	0.06***[0.06–0.06]
Birth order	-0.43***[-0.45–0.41]	- -	-0.43***[-0.45–0.41]
Number of ANC visits	0.03***[0.02–0.03]	- -	0.03***[0.02–0.03]
Mother’s years of education	0.01***[0.00–0.01]	0.03***[0.03–0.04]	0.04***[0.04–0.05]
Wealth index	1.27***[1.04–1.50]	7.29***[6.94–7.64]	8.56***[8.16–8.96]
**Weight at birth**			
2500–3999 g^®^			
<2500 g	0.21***[0.17–0.25]	- -	0.21***[0.17–0.25]
>4000 g	0.46***[0.39–0.54]	- -	0.46***[0.39–0.54]
Not weighted	-0.22***[-0.29–0.15]	- -	-0.22***[-0.29–0.15]
**Health insurance**			
No^®^			
Yes	0.08***[0.04–0.12]	-0.03 [-0.09–0.02]	0.05 [-0.02–0.12]
**Place of residence**			
Rural^®^			
Urban	0.09***[0.06–0.13]	0.17***[0.13–0.22]	0.26***[0.21–0.32]
**Caste group**			
Others^®^			
Other Backward Class (OBC)	-0.31***[-0.35–0.28]	0.16***[0.12–0.21]	-0.15***[-0.21–0.09]
Scheduled Caste (SC)	-0.19***[-0.23–0.14]	-0.24***[-0.30–0.18]	-0.43***[-0.50–0.35]
Scheduled Tribe (ST)	-0.51***[-0.56–0.46]	-0.41***[-0.49–0.34]	-0.92***[-1.01–0.83]
**Breech presentation**			
No^®^			
Yes	0.09***[0.04–0.13]	- -	0.09***[0.04–0.13]
**BMI**			
Normal^®^			
Underweight	-0.22***[-0.26–0.18]	- -	-0.22***[-0.26–0.18]
Overweight	0.51***[0.47–0.55]	- -	0.51***[0.47–0.55]
Obese	0.89***[0.83–0.96]	- -	0.89***[0.83–0.96]
**Place of ANC visits**			
No ANC/at home^®^			
Only public hospitals	0.19***[0.14–0.24]	-1.42***[-1.49–1.36]	-1.23***[-1.31–1.16]
Only private hospitals	0.09***[0.04–0.14]	2.25***[2.17–2.33]	2.34***[2.25–2.43]
Public & private hospitals	0.31***[0.25–0.38]	0.78***[0.71–0.85]	1.09***[1.00–1.19]
**Regions**			
North India^®^			
South India	0.68***[0.63–0.72]	0.36***[0.30–0.42]	1.04***[0.96–1.11]
West India	-0.18***[-0.23–0.12]	1.02***[0.94–1.09]	0.84***[0.75–0.93]
Central India	-0.06**[-0.11–0.00]	-0.88***[-0.96–0.81]	-0.94***[-1.04–0.85]
East India	0.26***[0.21–0.30]	-0.02 [-0.07–0.04]	0.24*** [0.17–0.31]
North-east India	0.34***[0.29–0.40]	-0.36***[-0.44–0.29]	-0.02[-0.11–0.07]
***Endogenous variable***			
**Place of delivery**			
Public hospitals^®^			
Private hospitals	1.31***[1.27–1.34]	- -	1.31***[1.27–1.34]

^**®**^ = Reference category;

*** significant level at <0.0001,

** significant level at <0.0100, and

* significant level at <0.0500; B = Unstandardised coefficient; CI = Confidence Interval.

**Source**: Computed from the National Family Health Survey (NFHS-4), 2015–16.

Among other exogenous variables, the mother’s BMI, caste group, birth weight, place of residence and geographical region are the important factors influencing CS delivery directly and/or indirectly or both the way in the path models. As compared to others or the general category, that is, the socially advanced community, all the categories of socially deprived communities have negative total effects on CS delivery (OBC, B = -0.15, *p*<0.0001; SC, B = -0.43, *p*<0.0001; ST, B = -0.92, *p*<0.0001). However, surprisingly only OBCs have a positive relationship with the place of delivery. In comparison to rural residence, urban residence has a positive total effect on CS delivery, although its influence largely passes indirectly through place of delivery. The indirect effects and total effects of having health insurance on CS delivery are not significant, while a small positive direct effect is seen. The total effects and indirect effects of regions on CS delivery differ widely across the six major geographical regions. Among all the regions, southern India exhibits the highest positive total effect on CS delivery, followed by western India and eastern India, whereas central India shows a higher negative total effect on the same. A very striking finding is that indirect effect via place of delivery of western India (B = 1.02, *p*<0.0001) is higher than the total effect (B = 0.84, *p*<0.0001), while the direct effect is negative on the same. In the case of southern India, the indirect effect (B = 0.36, *p*<0.0001) is fairly lower than the total effect (B = 1.04, *p*<0.0001) while the direct effect (B = 0.63, *p*<0.0001) is higher than the indirect effect on CS delivery. On the other hand, central India shows its negative effect on CS delivery in all ways and most of them are explained by place of delivery.

## 4. Discussion

It has been found that the national CS delivery rate has become doubled over a decade from 2005–06 to 2015–16 in India. About 17 per cent of children were born by CS delivery among all live births, 19 per cent of children were born by CS delivery among the most recent live births, and 23 per cent of children were born by CS delivery among the most recent institutional live births, during the five years before the survey, 2015–16. Thus the decadal rise of CS rate and the rise of CS rate in between all live births and most recent live births evidence a steep increasing trend in CS delivery in India. It has also been noticed that there are about 87 per cent chances of having repeat CS if previous birth is CS. In contrast, only about 12 per cent chances of having VBAC. From the findings of the NFHS-4 conducted during 2015–16, it is seen that (Fig 3 & Table 1) about two children in five children are delivered by CS in private hospitals (42.7 per cent), whereas in public hospitals the level of CS deliveries is much lower, about one in eight (13.2 per cent). From this, one can presume that delivery in private hospitals is the principal factor contributing to CS deliveries. And the inferences based on multiple regressions also lead to a similar conclusion. However, the results of path analysis using generalised structural equation modelling show a more nuanced picture.

### 4.1. Tested hypothesised causal pathway

This study hypothesised that certain socio-economic characteristics (e.g. mother’s education, income) and institutional factors (e.g. place of ANC visits) would contribute to CS deliveries largely indirectly through the place of delivery. And the results of path analysis reinforced the hypotheses. From the results shown in Table 3, it was found that the wealth index, a proxy measure of household income, and the place of ANC visits influence CS deliveries largely through delivery taking place in private hospitals. The detailed results of this study have been described thematically below.

### 4.2. Socio-economic factors

The effect of income on CS delivery was almost six and a half times high that of the place of delivery, although most of the income effect was mediated by place of delivery, that is, delivery taking place in private hospitals. A large number of previous studies also recognised that the household or family income largely controls accessibility to private hospitals [24–28] because the costs for doctors, hospitalisation, etc. are quite high in private hospitals which everyone in India cannot afford. Thus, easy access to the costlier private hospitals for wealthier households, in turn, results in higher incidents of having a CS delivery. In this regard, a recent study conducted in India revealed that CS deliveries are higher among mothers who have a higher socio-economic status [11]. On the other hand, the effect of a mother’s education on CS delivery was found to be very small in the present analysis. In addition to the income and place of ANC services, the Indian social structure, as reflected in the caste system, played a crucial role in the mode of delivery. CS deliveries were negatively associated with deprived communities, i.e. OBCs, SCs and STs with respect to the non-deprived communities (others) or general category. It should be noted that these effects were seen even after the effects of other important socio-economic factors such as income and education were controlled in the regressions. This result is consistent with the result of another study conducted in India [23]. However, it should be said here that Other Backward Classes (OBC), a less deprived community, had a positive effect with accessibility to private hospitals and made a positive effect on CS deliveries indirectly, while membership of the relatively more deprived communities, i.e. SCs and STs, was negatively correlated with delivery in private hospitals. In India, it is a well-known fact that OBCs, SCs and STs are the socially and economically disadvantaged groups [55,56] and because of this, their accessibility to expensive private hospitals is lower for receiving ANC services and delivering babies, which might also be the cause of a lower rate of CS deliveries among them. Urban residence was positively correlated to deliveries in private hospitals and to CS deliveries. This echoes the findings of a study based on the Asian context [26]. In India, private hospitals are mostly located in urban areas, and thus are more easily accessible to the urban population than the rural.

### 4.3. Maternal and pregnancy-related factors

Maternal and pregnancy-related factors were not connected indirectly or through the place of delivery to the mode of delivery in the causal pathway diagram. These were directly linked to the mode of delivery. Among maternal and pregnancy-related factors, the mother’s body mass index (BMI), birth order and birth weight of the baby were the important factors of CS delivery. While the mother’s age at last birth, number of ANC visits and breech presentation during delivery were relatively less important factors influencing CS delivery. A positive association between the mother’s BMI and CS delivery was found. The same observation has been reported by other studies [18,39]. Obesity is associated with pregnancy complications, e.g. gestational diabetes, preeclampsia, induced labour [57,58] which may increase the chances of CS delivery. There was an inverse relationship between birth order and CS delivery. This finding concurs with the finding of other studies [11,41,42]. Pregnancy and delivery-related complications are higher among the primiparous women or women of lower birth order than women of higher birth order [59] which may lead to higher chances of CS delivery. A baby’s extreme birth weight (it could be below or above the normal range, 2500–3999 g), was positively correlated with CS delivery and a similar association is also observed in previous studies [18,43,60].

### 4.4. Institutional factors

The effect of receiving ANC services at only private hospitals on CS delivery was high, although most of it was mediated through place of delivery (in private hospitals). A previous study had also demonstrated that receiving ANC services in private hospitals leads to opting for delivery in private hospitals [29] and subsequently to a CS delivery [47,48]. This might probably be due to the development of a relationship between clients or pregnant mothers and private gynaecologists/obstetricians during the pregnancy check-ups, which encourages mothers to be delivered by the same doctor or in the same hospital, resultant it has a higher chance of CS delivery as a private hospital has its role in performing CS delivery. A lower-level direct positive effect of health insurance coverage on CS delivery was found. A similar association was also found in other studies [49,50]. On the other hand, health insurance coverage did not show any significant effect on the place of delivery in the path models, while an earlier study had shown a positive association between having health insurance and delivery in private hospitals [29].

### 4.5. Spatial factors

Another important factor influencing CS delivery was geographical region. Wide variations in CS delivery across the six major geographical regions were found, and a complex phenomenon was also observed between direct effects, indirect effects and total effects. In comparison to northern India, southern India followed by western India have shown higher positive effects on CS delivery. Unlike southern India, western India showed a negative direct effect on CS delivery and a higher positive indirect effect based on place of delivery. The possible explanation is that the highest proportion of almost 53 per cent (S1 Table) deliveries took place in private hospitals in western India. Thus the positive effect on CS deliveries has largely been through the place of delivery rather than the region. In the case of southern India, however, the direct positive regional effect on CS delivery was observed, and that was higher than the indirect effect, even though about 46 per cent deliveries took place in private hospitals in this region. A possible reason is that southern India’s socio-economic conditions and health infrastructure are relatively better than those in western India and the other parts of the country [61] and thus the population in this region has easier access to private health facilities thereby enhancing the rate of CS deliveries. Further, the effect of health insurance is captured in the path models and the insurance coverage is higher in this region as compared to other regions, which might be another reason for higher CS deliveries here. On the other hand, central India showed a high negative effect on CS delivery, and the place of delivery. The probable reasons might be because it lies at the lowest level of development in terms of health, education and infrastructure [61] and therefore a very low proportion of about 17 per cent deliveries took place in private hospitals in central India. Further, it was found that the indirect effects were negative on CS delivery for eastern and north-eastern India respectively. However, they showed some positive direct effects on CS delivery. Accessibility to private hospitals was significantly lower in central India, eastern and north-eastern India than the other regions, which was also revealed by a study conducted in India [25]. In order to investigate the complex phenomena of whether deliveries taking place in private hospitals or other factors make differences in CS deliveries across the regions, further comparative studies among the regions or states are needed.

### 4.6. Place of delivery

The place of delivery was considered as an endogenous variable in the hypothesised causal path model. Expectedly, the place of delivery acted as a mediator and played a pivotal role in CS deliveries in this study. Although the individual effect of place of delivery was lower than that of income and receiving ANC services at only private hospitals, it had a higher level of effect on CS deliveries after adjusting other factors. Moreover, it should be mentioned here that the direct effect of place of delivery is still the highest in the path models. In this regard, there are a large number of studies that also showed that place of delivery has a high effect on CS delivery [14,16,19,21,23]. The place of delivery would have shown an even greater effect on CS delivery if inferences had been based on logistic regression (S2 Table). But, in the path models, it is seen that income (wealth index) and receiving ANC services at only private hospitals influence CS deliveries largely through the place of delivery. From this finding, it could be said that the place of delivery is a proximate determinant of CS delivery or a mediator of other co-factors rather than a stronger predictor of it. The higher rate of CS deliveries in private hospitals could be explained in various ways. Firstly, the proprietors of private health facilities are revenue-oriented [17,49,50,62], and they try to encourage doctors to perform CS deliveries instead of normal deliveries because the former brings more revenue. Secondly, many doctors are also financially motivated and, therefore, advise patients to have a CS delivery. Thirdly, usually, doctors are very busy persons, engaged in multiple tasks, thus often they perform CS deliveries even before the arrival of labour pains to avoid a patient’s call at odd hours. Fourthly, both doctors and proprietors of private health facilities do not want to take risks [22] regarding a delivery and would not want to be blamed if a vaginal delivery results in permanent harm to mother or baby or maternal or foetal death. Lastly, the higher incidence of CS delivery in private hospitals could also be explained by maternal demand for CS and because of having a previous CS delivery [63]. The key limitations of this study are: (i) information on whether CS delivery was performed on maternal request was not in the study dataset, thus this study was unable to control the effect of maternal demand for CS delivery in the analysis; (ii) this study lacks medical/obstetrical knowledge that could lose the reading interest of a medical person.

## 5. Conclusions

The present study raised the question on the basis of findings of previous studies reviewed in the introductory section, why do private hospitals contribute largely to CS deliveries over public hospitals in developing countries. Is it the case that delivery in a private hospital contributes largely to a Caesarean birth? And then, what are the factors influencing the childbirth at a private hospital? In furtherance of that, a conceptual framework was developed and the generalised structural equation modelling (GSEM) technique was adopted for the analysis. To test the hypotheses, this study analysed 146,280 most recent live births delivered in hospitals only during the five years preceding India’s Fourth National Family Health Survey (NFHS-4), carried out from 2015 to 2016.

The results revealed that the overall institutional CS delivery rate for the last births was 23.4 in India. Of this percentage, 63.3 per cent and 36.7 per cent occurred in private and public hospitals respectively. The results of path analyses established that all the exogenous variables with the endogenous ones considered in the path models were based on the survey of literature influencing the chances of CS delivery, which could be directly and/or indirectly or both ways. Further, the results reinforced the hypotheses and established the fact that wealth index, a proxy measure of a household’s income, and the place of ANC visits influence CS delivery largely through the delivery taking place in a private hospital. Furthermore, it was revealed that the place of delivery is a proximate determinant of CS delivery or a mediator of other co-factors rather than a stronger predictor of it. Many sections of society, especially economic well-off families prefer private health institutions over public ones because of their perception of the quality of services in the public health sector.

Improving the overall accessibility and quality of public health facilities is likely to promote a greater use of these and, in turn, prevent the higher risk of CS deliveries. In spite of that, a high-level individual effect of place of delivery (in private hospitals) on CS delivery has been observed after taking into account other factors. Thus, universal guidelines, protocols and medical audits on CS deliveries should be implemented; the infrastructure and the quality of public health facilities need to be improved to attract mothers for delivery care; public health systems should encourage mothers to give childbirth in public hospitals and should advise not going for CS delivery if it is non-medically indicated; public health systems should also instruct private health facilitators or doctors to avoid medically unnecessary CS delivery; and in the case of maternal request for a CS delivery, doctors should be directed to aware them about the adverse impacts of CS delivery. According to Arjun (2008) [64], women should be well informed of their fundamental right to deliver a baby vaginally, and they must be told that a CS delivery does not automatically protect newborn and maternal health [64]. These may help to reduce excess CS deliveries. In addition to income, place of ANC services and place of delivery, Indian caste group systems played a crucial role in the mode delivery. The mother’s BMI was also an important factor of CS delivery in this study. So, universal health education needs to be introduced to make mothers aware about the benefits and problems, and the risk factors of a CS delivery, which may also help to reduce the higher rate of CS deliveries. Recently, an evaluation study based in China published in a reputed journal reveals that the incidence of CS deliveries has been declined after the implementation of a two-stage intervention package [65]. Similar kind of intervention needs to be introduced in India as well as in other developing countries to reduce CS deliveries. Also, non-clinical interventions at pregnant mothers, health professionals, and health care services may reduce CS deliveries [66,67]. Besides, wide variations in CS deliveries across the six major geographical regions were found, and a complex phenomenon was also observed between direct effects, indirect effects and total effects of regions. Further studies are needed to investigate this complex phenomenon and to understand the inequalities in CS deliveries across the regions as well.

## Supporting information

S1 Table Proportion distribution of delivery in private and public hospitals by regions, India (NFHS-4). (DOCX)

S2 Table Estimated odds ration using binary logistic regression for CS delivery. (DOCX)
