# Supplementary material for: Does delivery in private hospitals contribute largely to Caesarean Section births? A path analysis using generalised structural equation modelling
Source: PLoS One. 2020 Oct 8;15(10):e0239649. doi: 10.1371/journal.pone.0239649 (PMC7544137; doi:10.1371/journal.pone.0239649)
Supplement: S1 Table — (DOCX) [file pone.0239649.s001.docx]

| **Supplementary Table 1. Proportion distribution of delivery in private and public hospitals by regions, India (NFHS-4)** | | |
| --- | --- | --- |
| **Regions** | **Private hospitals** | **Public hospitals** |
| North India | 32.9 | 67.1 |
| South India | 45.8 | 54.2 |
| West India | 52.5 | 47.5 |
| Central India | 16.9 | 83.1 |
| East India | 24.5 | 75.5 |
| North-east India | 17.2 | 82.7 |
| **India** | **34.7** | **65.3** |
| **Source:** Computed from the National Family Health Survey (NFHS-4), 2015‒16 | | |
