# Supplementary material for: Does delivery in private hospitals contribute largely to Caesarean Section births? A path analysis using generalised structural equation modelling
Source: PLoS One. 2020 Oct 8;15(10):e0239649. doi: 10.1371/journal.pone.0239649 (PMC7544137; doi:10.1371/journal.pone.0239649)
Supplement: S2 Table — (DOCX) [file pone.0239649.s002.docx]

| **Supplementary Table 2. Estimated odds ration using binary logistic regression for CS delivery** | | | |
| --- | --- | --- | --- |
| **Variables** | **Odds Ratio** | ***p*-value** | **[95% Conf. Interval]** |
| ***Continuous variables*** |  |  |  |
| Mother’s age | 1.06 | <0.0001 | 1.06-1.07 |
| Birth order | 0.65 | <0.0001 | 0.64-0.66 |
| Number of ANC visits | 1.03 | <0.0001 | 1.03-1.03 |
| Mother’s year of education | 1.01 | <0.0001 | 1.00-1.01 |
| Wealth index | 1.00 | <0.0001 | 1.00-1.00 |
| ***Categorical variables*** |  |  |  |
| **Weight at birth** |  |  |  |
| 2500-3999 g® |  |  |  |
| <2500 g | 1.24 | <0.0001 | 1.19-1.29 |
| >4000 g | 1.59 | <0.0001 | 1.48-1.71 |
| Not weighted | 0.80 | <0.0001 | 0.75-0.86 |
| **Health insurance** |  |  |  |
| No® |  |  |  |
| Yes | 1.09 | <0.0001 | 1.04-1.13 |
| **Place of residence** |  |  |  |
| Rural® |  |  |  |
| Urban | 1.10 | <0.0001 | 1.06-1.14 |
| **Caste group** |  |  |  |
| Others® |  |  |  |
| Other Backward Class (OBC) | 0.73 | <0.0001 | 0.70-0.76 |
| Scheduled Caste (SC) | 0.83 | <0.0001 | 0.79-0.87 |
| Scheduled Tribe (ST) | 0.60 | <0.0001 | 0.57-0.63 |
| **Breech presentation** |  |  |  |
| No® |  |  |  |
| Yes | 1.09 | <0.0001 | 1.06-1.14 |
| **BMI** |  |  |  |
| Normal® |  |  |  |
| Underweight | 0.80 | <0.0001 | 0.77-0.83 |
| Overweight | 1.66 | <0.0001 | 1.60-1.73 |
| Obese | 2.45 | <0.0001 | 2.29-2.61 |
| **Place of ANC visits** |  |  |  |
| No ANC/at home® |  |  |  |
| Only public hospitals | 1.21 | <0.0001 | 1.15-1.26 |
| Only private hospitals | 1.09 | <0.0010 | 1.04-1.15 |
| Public & private hospitals | 1.37 | <0.0001 | 1.28-1.46 |
| **Regions** |  |  |  |
| North India® |  |  |  |
| South India | 1.97 | <0.0001 | 1.88-2.06 |
| West India | 0.84 | <0.0001 | 0.79-0.88 |
| Central India | 0.94 | <0.0340 | 0.89-1.00 |
| East India | 1.29 | <0.0001 | 1.23-1.35 |
| North-east India | 1.41 | <0.0001 | 1.34-1.49 |
| **Place of delivery** |  |  |  |
| Public hospitals® |  |  |  |
| Private hospitals | 3.70 | <0.0001 | 3.57-3.84 |
| **®**=Reference category |  |  |  |
| **Source:** Computed from the National Family Health Survey (NFHS-4), 2015‒16 | | | |
